# Heterologous Expression of Chrysanthemum TCP Transcription Factor CmTCP13 Enhances Salinity Tolerance in *Arabidopsis*

**DOI:** 10.3390/plants13152118

**Published:** 2024-07-31

**Authors:** Xinran Chong, Yanan Liu, Peiling Li, Yue Wang, Ting Zhou, Hong Chen, Haibin Wang

**Affiliations:** 1 Jiangsu Key Laboratory for the Research and Utilization of Plant Resources, Institute of Botany, Jiangsu Province and Chinese Academy of Sciences, Nanjing Botanical Garden Mem. Sun Yat-Sen, Nanjing 210014, China; 2 Institute of Jiangxi Oil-Tea Camellia, Jiujiang University, Jiujiang 332005, China; 3 College of Horticulture, Xinyang Agriculture and Forestry University, Xinyang 464000, China; 4 State Key Laboratory of Crop Genetics and Germplasm Enhancement, Key Laboratory of Landscaping, Ministry of Agriculture and Rural Affairs, College of Horticulture, Nanjing Agricultural University, Nanjing 210095, China

**Keywords:** TCP transcription factor, *Chrysanthemum morifolium*, salinity, overexpression, expression analysis

## Abstract

Plant-specific TEOSINTE BRANCHED1/CYCLOIDEA/PROLIFERATING CELL FACTOR (TCP) proteins play critical roles in plant development and stress responses; however, their functions in chrysanthemum (*Chrysanthemum morifolium*) have not been well-studied. In this study, we isolated and characterized the chrysanthemum TCP transcription factor family gene *CmTCP13*, a homolog of *AtTCP13*. This gene encoded a protein harboring a conserved basic helix–loop–helix motif, and its expression was induced by salinity stress in chrysanthemum plants. Subcellular localization experiments showed that CmTCP13 localized in the nucleus. Sequence analysis revealed the presence of multiple stress- and hormone-responsive *cis*-elements in the promoter region of *CmTCP13*. The heterologous expression of *CmTCP13* in *Arabidopsis* plants enhanced their tolerance to salinity stress. Under salinity stress, *CmTCP13* transgenic plants exhibited enhanced germination, root length, seedling growth, and chlorophyll content and reduced relative electrical conductivity compared with those exhibited by wild-type (WT) plants. Moreover, the expression levels of stress-related genes, including *AtSOS3*, *AtP5CS2*, *AtRD22*, *AtRD29A*, and *AtDREB2A*, were upregulated in *CmTCP13* transgenic plants than in WT plants under salt stress. Taken together, our results demonstrate that *CmTCP13* is a critical regulator of salt stress tolerance in plants.

## 1. Introduction

Plants are often subjected to various environmental stresses throughout their life cycle. Soil salinity is a major stress factor affecting plant growth, development, and productivity. It causes osmotic stress, ion imbalance, and oxidative toxicity [1,2,3]. Improving the salt tolerance of plants has become crucial given the increasing soil salinization worldwide. Understanding the mechanisms underlying plant responses to salinity stress is a prerequisite for identifying potential genes that can be used to improve genetic salt tolerance in plants.

Plants adapt to salt stress through various responses, including stress perception, signal transduction, and the expression of stress-related genes and metabolites [4]. Many transcription factors (TFs), such as DREB/CBF, MYB, MYC, and AREB/ABF, play crucial roles in stress responses by regulating the expression of downstream target genes [5]. Overexpression of certain TFs, such as DREB2A and ERF, can enhance plant tolerance to various abiotic stresses including salinity [6,7]. The TCP family is a group of plant-specific TFs named after three initially characterized members, i.e., TEOSINTE BRANCHED 1 (TB1), CYCLOIDEA (CYC), and PROLIFERATING CELL FACTORS (PCFs). These proteins harbor a 59 amino acid noncanonical basic helix–loop–helix (bHLH) motif known as the TCP domain [8]. TCP proteins consist of 24 members in *Arabidopsis thaliana* [9], 22 in *Oryza sativa* [10], 33 in *Populus euphratica* [11], and 66 in *Triticum aestivum* [12]. Based on the dissimilarities in TCP domains, *TCP* genes can be classified into two classes, class I (known as PCF or TCP-P) and class II (TCP-C), which are further subdivided into two subclades, CYC/TB1 and CINCINNATA (CIN) [13,14].

TCP proteins play diverse and critical roles in many plant-specific biological processes including seed germination [15], leaf development [16], shoot branching [17], flower development [18,19], circadian rhythms [20], and hormonal pathways [9]. In addition, many TCP family genes coordinate plant responses to environmental stress [8,21]. In rice, OsPCF2 binds directly to the promoter of the *O. sativa* gene Na^+^/H^+^ exchanger 1 (*OsNHX1*) to upregulate its expression and enhance salt tolerance [22]. The *OsTCP19* gene reportedly affects salt and drought stress tolerance positively [23]. Moreover, 46 *ZmTCP* genes have been identified in maize (*Zea mays*), and only *ZmTCP42*, which plays a positive role in drought tolerance, has been well characterized [24]. In Moso bamboo (*Phyllostachys edulis*), *PeTCP10* can be induced by salt stress, and its overexpression enhances the salt tolerance of transgenic *Arabidopsis* at the vegetative growth stage [25]. Recently, *BpTCP20* was reported to confer salt tolerance through the acetyltransferase component of the pyruvate dehydrogenase complex (BpPDCE23)-mediated regulation of acetylation [26].

Chrysanthemum (*Chrysanthemum morifolium*) is a species of global significance with high ornamental, cultural, and economic value. Salinity stress is a major factor affecting the yield and quality of chrysanthemum and can lead to extensive leaf chlorosis, growth retardation, and, in a few cases, even plant death. Therefore, increasing the salinity tolerance of chrysanthemum plants is essential for achieving stable and sustainable production. Recently, the reference genome of *C. morifolium* was reported, which laid the foundation for the genetic and molecular breeding of chrysanthemum [27]. Hitherto, there have been limited studies on the functions of TCP proteins in chrysanthemums. Therefore, in this study, we isolated a chrysanthemum TCP family TF gene, *CmTCP13*, and showed that its expression was induced by salinity stress. Overexpression of *CmTCP13* in *Arabidopsis* significantly enhanced the salinity tolerance of transgenic plants. Through this study, we provide an excellent candidate gene for genetically improving salt tolerance in chrysanthemum plants.

## 2. Results

### 2.1. Cloning and Sequence Analysis of CmTCP13

The TCP TF family gene *CmTCP13* was cloned from the chrysanthemum cultivar ‘Jinba’. The gene consisted of a 1032 bp open reading frame (ORF) encoding a 343 amino acid protein with a predicted molecular weight of 38.42 kDa and an isoelectric point of 6.63.

**Figure 1:**
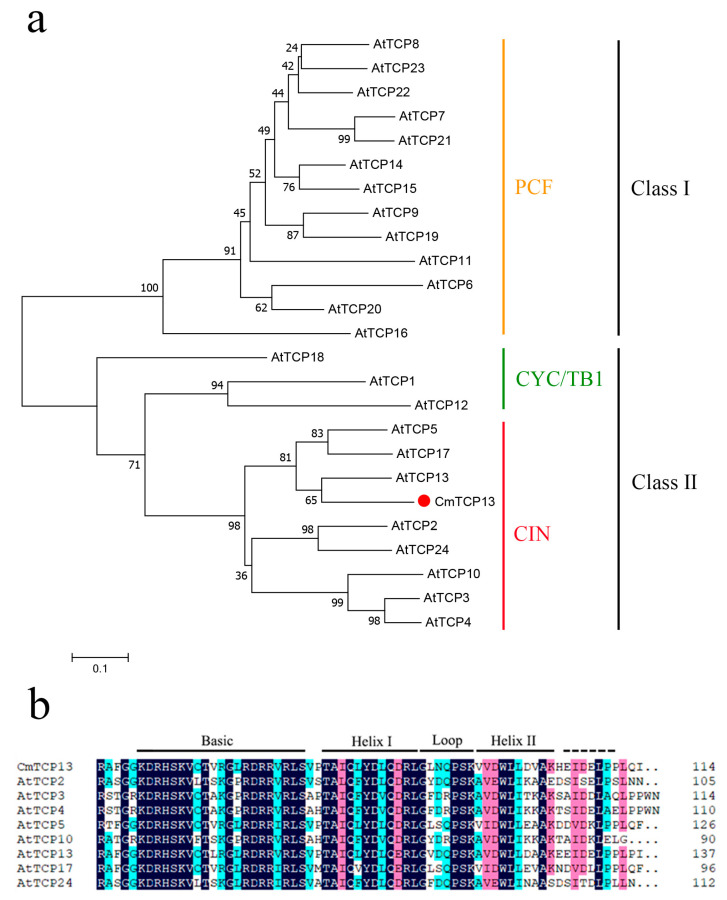
Characterization of the CmTCP13 polypeptide sequence: (**a**) Phylogenetic tree of CmTCP13 and other *Arabidopsis* TCP proteins; (**b**) Alignment of the amino acid sequences of the bHLH domain. The conserved motifs are highlighted in black lines.

The phylogenetic analysis of CmTCP13 with 24 members of the *Arabidopsis* TCP family revealed that CmTCP13 is most closely related to AtTCP13 and belongs to the CIN subclass of Class II TCP proteins (Figure 1a). In addition, multiple sequence alignment of CmTCP13 with *Arabidopsis* CIN TCPs revealed that CmTCP13 harbors a conserved bHLH motif in its N-terminal region (Figure 1b).

### 2.2. Subcellular Localization of CmTCP13

**Figure 2:**
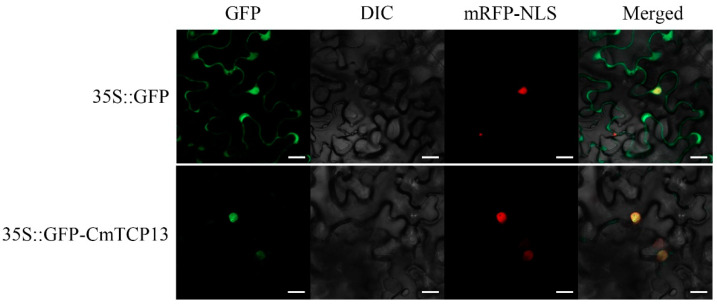
Subcellular localization of CmTCP13 in tobacco epidermal cells. Green fluorescent protein (GFP): Images under the green fluorescence channel; Differential interference contrast (DIC): Images under bright light; Red fluorescent protein with a nuclear localization signal (mRFP-NLS): D53-mCherry was used as a nuclear marker; Merged: overlay plots. Scale bars represent 20 μm.

The *35S::GFP* control vector and *35S::GFP-CmTCP13* fusion construct were transiently transformed into the epidermal cells of *Nicotiana benthamiana* together with the 35S::D53-RFP construct as a nuclear marker to explore the subcellular localization of the CmTCP13 protein. The GFP signal of the control *35S::GFP* transgene was detected in both the cytoplasm and nucleus of the leaf epidermal cells, whereas the GFP fluorescence of the *35S::GFP-CmTCP13* transgene was specifically detected in the nucleus and co-localized with the nuclear marker D53-mCherry (Figure 2). These results indicate that *CmTCP13* is localized in the nucleus.

### 2.3. Transcript Profile of CmTCP13 in Response to Salinity

**Figure 3:**
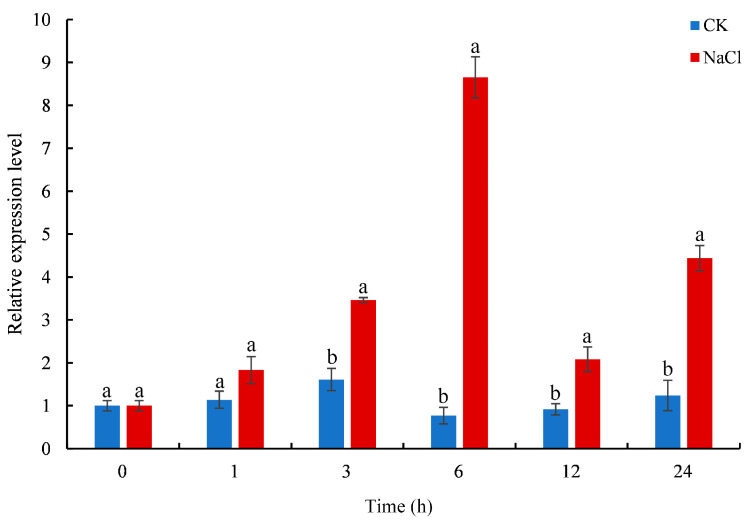
Expression pattern of *CmTCP13* in chrysanthemum ‘Jinba’ under salinity treatment as assayed by qRT-PCR. Values represent means ± standard deviation (SD) with three biological replicates. Letters above bars indicate statistically significant differences between the control (CK) and NaCl treatment (*p* < 0.05).

The expression levels of *CmTCP13* in chrysanthemum ‘Jinba’ under salinity stress conditions were evaluated by quantitative real-time polymerase chain reaction (qRT-PCR). *CmTCP13* transcript levels increased considerably and peaked at 6 h after salt treatment. Subsequently, *CmTCP13* expression levels decreased but remained significantly (*p* < 0.05) higher than those of the untreated control, showing a 3.58-fold increase at 24 h after the treatment (Figure 3). This finding indicates that *CmTCP13* may be involved in regulating the salt stress response.

### 2.4. Cis-Acting Regulatory Element Analysis of CmTCP13 Promoter Sequence

We cloned the 2405 bp promoter sequence upstream of *CmTCP13* and analyzed its *cis*-acting elements using the PlantCARE database. The *CmTCP13* promoter contained multiple hormone-responsive elements, such as abscisic acid (ABA) response element (ABRE), methyl jasmonate (MeJA) response elements (CGTCA and TGACG motifs), ethylene response element (ERE), auxin response elements (AuxRR-core and TGA-element), and gibberellin and salicylic acid (SA) response elements (P-box and TCA-element, respectively) (Table 1). In addition, certain *cis*-acting elements are induced by adversity stress, such as anaerobic responsive element (ARE), low-temperature response (LTR) element, MYB binding site (MBS), wound-responsive (WUN) motif, GT-1 motif, and MYB and MYC recognition sites, among which the GT-1 motif is a salinity stress-inducible element (Table 1). Moreover, the *CmTCP13* promoter contained light-responsive elements, including Box 4, G-box, I-box, GA-motif, and GATA-motif, and a circadian rhythm regulatory *cis*-element (circadian) (Table 1). These results indicate that *CmTCP13* is widely involved in plant responses to hormones and abiotic stress.

### 2.5. CmTCP13 Overexpression in Arabidopsis Enhanced Salinity Tolerance

**Figure 4:**
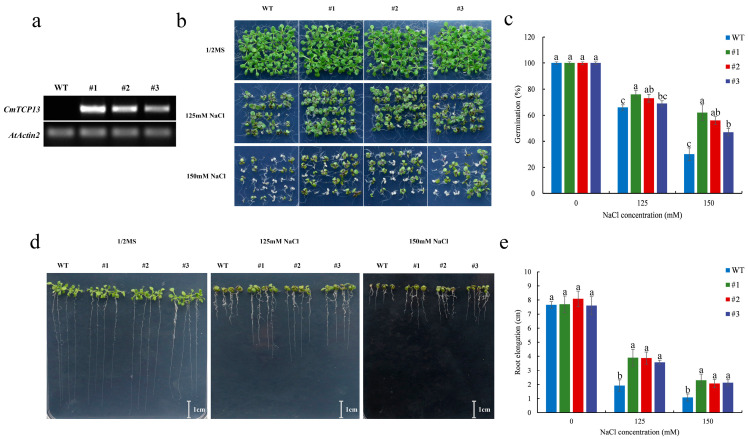
Phenotypic comparison of the growth of the WT and *CmTCP13*-expressing transgenic *Arabidopsis* plants under salt stress. (**a**) RT-PCR analyses of *CmTCP13* expression in WT and T_3_ transgenic plants. *AtActin2* was the endogenous reference gene. (**b**,**c**) Germination of WT and transgenic seeds grown for 10 days on 1/2MS medium containing 125 and 150 mM NaCl. (**d**,**e**) Root elongation of WT and transgenic plants after 9 days of growth on 1/2MS medium with different NaCl concentrations. Data represent means ± standard error (SE), and different letters indicate significant differences between WT and transgenic plants at *p* < 0.05, as determined by Duncan’s test.

To explore the function of *CmTCP13* in *Arabidopsis*, three transgenic lines from the T_3_ generation were selected. *CmTCP13* transgene transcription was detected in the transgenic lines (#1, #2, and #3) but not in wild-type (WT) plants (Figure 4a). In addition, we also explored the expression levels of *Arabidopsis AtTCP13* in *CmTCP13* transgenic *Arabidopsis* lines. The results showed no significant change in the expression levels of endogenous *AtTCP13* in the transgenic plants relative to those in WT plants (Figure S1). Salinity stress tolerance was compared between the three T_3_ transgenic lines and WT plants. No apparent phenotypic differences were observed when seedlings were germinated on a half-strength Murashige and Skoog (1/2MS) medium. However, when these lines were germinated on 1/2MS medium containing 125 and 150 mM NaCl, the seeds from the three transgenic lines showed higher germination than that of WT seeds (Figure 4b,c). On the 150 mM NaCl medium, WT seed germination was only 30.00%, whereas that of the #1, #2, and #3 transgenic line seeds was 62.22, 55.56, and 46.67%, respectively (Figure 4c). We further tested the root growth of the three transgenic lines and WT plants under salt stress. Root length between the transgenic and WT plants on 1/2MS medium did not differ; however, in the presence of 125 and 150 mM NaCl, the transgenic lines exhibited a considerable increase in root length compared with that of WT plants (Figure 4d,e).

**Figure 5:**
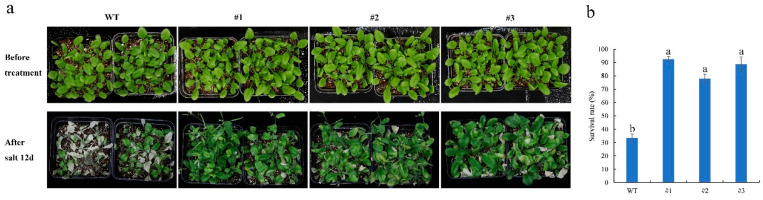
Salinity tolerance of the WT and *CmTCP13*-expressing transgenic *Arabidopsis* plants. (**a**) Phenotype of WT and *CmTCP13* overexpressing seedlings after irrigation with increasing NaCl concentrations. (**b**) Survival rates of the WT and *CmTCP13* transgenic plants under salt stress. Values are presented as means ± SE (n = 45), and statistical differences are determined using Duncan’s test (*p* < 0.05).

In addition, for the salinity tolerance assay, 3-week-old WT and transgenic plants were sequentially watered with 100, 200, and 300 mM NaCl at 4-day intervals. After the salinity treatment, the three transgenic lines exhibited relatively lower sensitivity to salt stress (Figure 5a). The percentage survival of the #1, #2, and #3 *CmTCP13* transgenic lines was 96.30, 92.59, and 88.89%, respectively, which was significantly (*p* < 0.05) higher than that of the WT plants (29.63%) (Figure 5b). These results indicate that *CmTCP13* overexpression enhances salinity tolerance in *Arabidopsis*.

**Figure 6:**
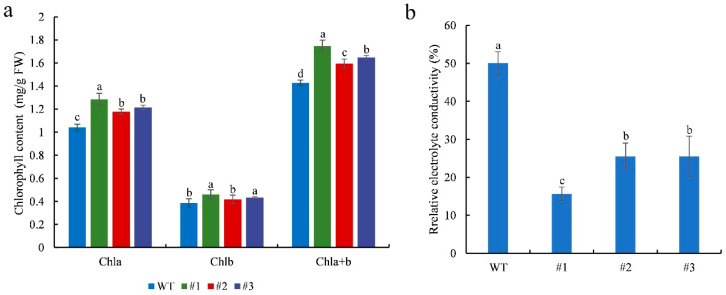
Physiological effects of salinity treatment on the WT and *CmTCP13*-expressing transgenic *Arabidopsis* plants: (**a**) Leaf chlorophyll content; FW, fresh weight; Chla, chlorophyll a content; Chlb, chlorophyll b content; Chla+b, total chlorophyll content. (**b**) Leaf relative electrolyte conductivity. Data represent means ± SE of three replicates, and different letters indicate significant differences between WT and the three transgenic lines at *p* < 0.05, as determined by Duncan’s test.

Chlorophyll content and electrolyte leakage were measured in WT and transgenic plants after 7 days of salinity stress. The leaf chlorophyll content of the treated transgenic plants was significantly (*p* < 0.05) higher than that of the treated WT plants (Figure 6a). These results indicate that *CmTCP13* transgene overexpression delays chlorophyll degradation. In contrast, relative electrolyte conductivity was significantly (*p* < 0.05) lower in transgenic plants than in WT plants (Figure 6b), indicating that *CmTCP13* overexpression protected plant membranes from damage under salinity stress.

### 2.6. CmTCP13 Overexpression Altered the Expression of Stress-Responsive Genes

**Figure 7:**
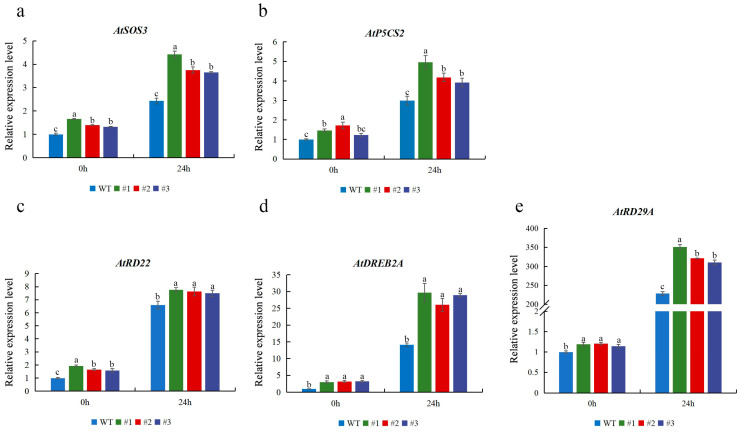
Expression of stress-related genes *AtSOS3* (**a**), *AtP5CS2* (**b**), *AtRD22* (**c**), *AtDREB2A* (**d**), *AtRD29A* (**e**), in the WT and *CmTCP13*-expressing transgenic *Arabidopsis* plants. Values represent means ± SE of three replicates, and significant differences between WT and transgenic plants are determined by Duncan’s test (*p* < 0.05).

To better elucidate the possible role of *CmTCP13* in response to salt stress, the expression of several stress-related genes was studied in *CmTCP13* transgenic lines and WT plants. When subjected to salinity stress, the expression of ion homeostasis-related genes, including *AtSOS3*, was upregulated in the transgenic plants (Figure 7a). The expression levels of *AtP5CS2*, which was implicated in osmotic regulation, were also significantly induced in the transgenic lines compared with those in WT plants (Figure 7b). In addition, the expression levels of other stress-responsive genes, including *AtRD22*, *AtRD29A*, and *AtDREB2A*, were significantly upregulated in the transgenic plants under salt stress (Figure 7c–e). These data indicated that *CmTCP13* may enhance salinity tolerance in *Arabidopsis* by inducing the expression of salt stress-related genes.

## 3. Discussion

Plant-specific TCP proteins are involved in many processes of plant growth and development [8,28]. Furthermore, *TCP* genes have been identified and characterized in various plant species, including *Arabidopsis*, *P. euphratica*, *Solanum lycopersicum*, and *Sorghum* [11,14,29,30]. However, few studies have reported the functions of TCP family members in chrysanthemum plants. The *CmTCP20* gene is reportedly involved in regulating petal elongation and root development in chrysanthemum [31,32]. The heterologous expression of *CmTCP14* from chrysanthemum suppresses organ size and delays senescence in *Arabidopsis* [33]. Hitherto, no studies have investigated the role of *TCP* genes in abiotic stress resistance regulation in chrysanthemum. In this study, we isolated a TCP family member *CmTCP13* with a conserved noncanonical bHLH motif in the N-terminal region. This domain has been implicated in DNA binding, nuclear targeting, and protein–protein interactions [8,34,35]. Phylogenetic analysis revealed that CmTCP13 belongs to the class II CIN TCP family and is most closely related to AtTCP13. A previous study showed that *AtTCP13* exhibits stress-inducible expression and plays a key role in regulating plant growth in leaves and roots under dehydration stress conditions [36]. Here, we found that *CmTCP13* was significantly induced by salt stress, similar to *PeTCP10* in *Phyllostachys edulis* [25] and *GbTCP4* in *Gossypium barbadense* [37]. Phylogenetic analysis and expression patterns indicated that *CmTCP13*, a TF, may be involved in the salinity stress response in chrysanthemum plants.

To further elucidate the mechanisms governing the upstream transcriptional regulation of the *CmTCP13* gene, a 2405 bp promoter region upstream of the gene was isolated from chrysanthemum ‘Jinba’. The analysis of the promoter sequence has shown the existence of various hormone and stress response *cis*-elements, such as ABRE, CGTCA-motif, TGACG-motif, ERE, AuxRR-core, TCA-element, ARE, LTR, MBS, MYB, and GT-1 motif. ABRE is an ABA-response element. Notably, ABA regulates many aspects of plant growth and development and plays a crucial role in plant responses to drought, salt stress, and other adversity stresses [5,38,39]. Jasmonic acid is also an important plant growth regulator, which is implicated in plant growth and development and stress resistance [40,41]. The *CmTCP13* promoter region has two MeJA-responsive elements, i.e., the CGTCA and TGACG motifs. In addition, plant hormones such as auxin, ethylene, gibberellin, and SA have been reported to play critical roles in plant responses to biotic and abiotic stresses [42,43,44,45]. The *CmTCP13* promoter region also contained ethylene- (ERE), auxin-(AuxRR-core), gibberellin-(P-box), and SA-(TCA-element)-responsive elements. Of the other *cis*-elements, ARE is an anaerobically inducible element present in the promoter region of the maize *Adh* gene [46], LTR is a low-temperature response element, the WUN-motif is a trauma response element, and MBS and MYB are the binding elements of stress-related MYB proteins. The GT-1 motif with the consensus sequence GAAAAA was first identified in soybean as a salt- and pathogen-responsive element in the soybean calmodulin isoform-4 (*SCaM-4*) promoter. The interaction between the GT-1 motif and a GT-1-like TF plays a role in salt- and pathogen-induced *SCaM-4* gene expression in both soybean and *Arabidopsis* [47]. In the *CmTCP13* promoter region, we found seven salt-responsive GT-1 motifs, four of which were on the +ve DNA strand and three were on the −ve/complementary DNA strand (Table 1). The higher abundance of the salt-inducible GT-1 motif suggested that transcript expression of *CmTCP13* was highly induced by salinity stress.

In the present study, *Arabidopsis* plants overexpressing the *CmTCP13* transgene exhibited enhanced tolerance to salt stress. This result was supported by the higher germination, longer root lengths, and higher survival rates of the transgenic plants after salt treatment. Studies have reported that changes in physiology and biochemistry may be closely associated with resistance in transgenic plants [48]. The roots of the *CmTCP13* transgenic plants were longer than those of the WT plants, suggesting that *CmTCP13* may increase resistance by modulating the root system. Electrolyte leakage and chlorophyll content are commonly used indicators of plant membrane damage under salinity stress [1,5]. Relative leaf electrolyte leakage in the *CmTCP13* overexpressing plants was lower than in the WT plants under salinity stress, indicating that *CmTCP13* enhanced salinity tolerance by maintaining plant membrane integrity. Chlorophyllase activity was elevated under salt stress, leading to a decrease in chlorophyll content [49]. We found that the chlorophyll content of *CmTCP13* overexpressing plants was higher than that of WT plants under salinity stress, suggesting that *CmTCP13* might respond to salinity stress by regulating chlorophyll degradation. A higher chlorophyll content is associated with enhanced photosynthetic activity and increased disease resistance [50].

At the gene transcription level, the *CmTCP13* overexpressing plants exhibited an upregulation of stress-related genes, such as *AtSOS3*, *AtP5CS2*, *AtRD22*, *AtRD29A*, and *AtDREB2A*. The SOS3 protein is involved in the salt overly sensitive (SOS) signaling pathway in plants and is a key regulator of ion homeostasis and salt tolerance [51]. The overexpression of *SOS1* and *SOS3* increases salinity tolerance in transgenic *Arabidopsis* [52]. The SOS3 protein can interact with SOS2, and the SOS3/SOS2 kinase complex regulates the transport activity of SOS1, which encodes a plasma membrane Na^+^/H^+^ antiporter responsible for the exclusion of Na^+^ from cells [53,54]. The *AtP5CS* gene has been implicated in proline synthesis and transport in response to salt or osmotic stress [55,56]. The *RD29A* gene reportedly has a role in stress-related detoxification, thereby reducing stress-related injuries [57]. DREB proteins regulate the expression of several stress-responsive genes in response to biotic or abiotic stresses [58]. In our study, the increased expression of *CmTCP13* increased the transcript levels of salinity-induced stress response genes, including *AtSOS3*, *AtP5CS2*, *AtRD29A*, and *AtDREB2A*. Taken together, these results indicate that *CmTCP13* acts as a positive regulator of the salt response pathway by improving the expression of stress-responsive genes.

In summary, *CmTCP13*, a TCP family TF gene isolated from chrysanthemum, was significantly induced by salinity stress. *CmTCP13* overexpression in the transgenic *Arabidopsis* plants enhanced salt stress resistance compared with that in WT plants. These results suggest that *CmTCP13* positively regulates stress-related genes to enhance salt tolerance. In the future, the production of *CmTCP13*-overexpressing transgenic chrysanthemum plants will provide deeper insights into the mechanisms underlying the role of *CmTCP13* in salinity stress response.

## 4. Materials and Methods

### 4.1. Plant Materials and Salinity Stress Treatments

The chrysanthemum cultivar ‘Jinba’ was planted in the Nanjing Botanical Garden, Mem. Sun Yat-sen (118°49′55″ E, 32°3′32″ N), Nanjing, China. Uniformly rooted cuttings were grown in a 1:1 mix of garden soil and vermiculite and cultured in a greenhouse (day/night temperature: 25 °C/20 °C; photoperiod: 16 h; and relative humidity: 70%). Chrysanthemum seedlings were subjected to salinity stress at the 6–8 leaf stage by watering with 200 mM NaCl, whereas the control plants were treated with water (CK). We collected the third leaf from the apex of the experimental and control treatment plants (three plants each from both groups) at 0, 1, 3, 6, 12, and 24 h after salinity stress treatment. *A*. *thaliana* (ecotype Col-0) and transgenic plants were planted in a 1:3 mix of soilrite and vermiculite under a 16 h photoperiod with a day/night temperature of 22 °C/18 °C. After 3 weeks of growth, the WT and transgenic lines were subjected to 200 mM NaCl treatment, and three plants were harvested at 0 and 24 h after the salt treatment.

### 4.2. Isolation and Sequence Analysis of CmTCP13

Total RNA was isolated from chrysanthemum leaves using an RNA extraction kit (Huayueyang, Beijing, China), following the manufacturer’s protocol. Approximately 1 μg of total RNA was used to synthesize the first cDNA strand using SuperScript III Reverse Transcriptase (Invitrogen, Carlsbad, CA, USA). Based on the CL6155 contig 2 sequence in the chrysanthemum ‘Jinba’ transcriptome [59], the specific primer pair (*CmTCP13*-F/R; Table S1) was designed to amplify the *CmTCP13* coding sequence. To verify the *CmTCP13* nucleotide sequence, the PCR product was inserted into a pEASY-Blunt vector (TransGen Biotech, Beijing, China) for sequencing. The amino acid sequences of *Arabidopsis* TCP family members were acquired from the TAIR database (http://www.arabidopsis.org/, accessed on 16 July 2024), and a phylogenetic tree was constructed using the MEGA 7 software [60] based on the neighbor-joining algorithm with 1000 bootstrap replicates. Multiple sequence alignments of CmTCP13 and CIN AtTCP proteins were performed using the DNAMAN 6.0 software.

### 4.3. Subcellular Localization of CmTCP13

To generate the *35S::GFP-CmTCP13* fusion construct, the *CmTCP13* ORF (lacking the termination codon) was amplified using a KOD FX kit (Toyobo, Osaka, Japan) with the primer pair *CmTCP13*-R4-F/R (Table S1) harboring the *XhoI* and *SmaI* recognition sites. After purification, the amplicon was introduced into the pORE-R4 vector (35S::GFP) using the Gateway method, resulting in the plasmid *35S::GFP-CmTCP13*. The *Agrobacterium tumefaciens* strain *GV3101* carrying the pORE-R4 or pORE-R4-*CmTCP13* constructs was co-infiltrated with the p19 strain into the leaves of 5-week-old *Nicotiana benthamiana* as previously reported [61]. After culturing for 48–72 h at 22 °C, the GFP fluorescence signals were detected using a confocal laser scanning microscope (LSM 800, Carl Zeiss, Oberkochen, Germany).

### 4.4. Ectopic Expression of CmTCP13 in Arabidopsis

The *35S::CmTCP13* (pORE-R4-*CmTCP13*) construct was introduced into the *A. tumefaciens* strain EHA105 and then transformed into *A. thaliana* using the floral dip method [62]. Transformed progenies were selected using 1/2MS medium with 35 mg/mL kanamycin and advanced by self-pollination to obtain T3 transgenic plants. The T3 homozygous progenies were validated by RT-PCR using the primer pair *CmTCP13*-RT-F/R (Table S1). The expression of endogenous *AtTCP13* in the *CmTCP13* transgenic *Arabidopsis* lines was investigated by qRT-PCR using the primer pair *AtTCP13*-F/R (Table S1). The transcript levels of the *AtActin2* gene were used as a reference.

### 4.5. Cis-Acting Element Analysis in the Promoter Region

Genomic DNA was isolated from the fresh leaves of chrysanthemum ‘Jinba’ using a modified cetyltrimethylammonium bromide method [63]. The promoter fragment of *CmTCP13* was obtained from the chrysanthemum reference genome (http://210.22.121.250:8880/asteraceae/homePage, accessed on 12 June 2024), and full-length verification primers were designed to amplify the *CmTCP13* promoter from genomic DNA. The primer pair used (*CmTCP13*pro-F/R) is shown in Table S1. The *cis*-acting element analysis of the promoter sequence was performed using PlantCARE (http://bioinformatics.psb.ugent.be/webtools/plantcare/html/, accessed on 25 June 2024).

### 4.6. Salinity Tolerance Assay of Transgenic Arabidopsis Plants

For the germination assay, 30 seeds of the WT and *CmTCP13* overexpression plants each were sown on 1/2MS medium containing two different concentrations of NaCl, i.e., 125 and 150 mM. The germination was scored when the cotyledon remained green after 10 days. The assay was repeated thrice. For root length estimation, seeds were grown on 1/2MS medium plates placed vertically for 3 days and then transferred to 1/2MS medium plates containing the different NaCl concentrations (0, 125, and 150 mM). Root length was measured 9 days later. Three biological replicates were used for each experiment. In addition, for the salinity tolerance test, 90 plants each of 3-week-old WT and *CmTCP13* transgenic lines were watered for 12 days at 4-day intervals, first with 100 mM NaCl, then 200 mM NaCl, and finally with 300 mM NaCl, following Li et al. [64], and the survival rate was documented.

### 4.7. Measurements of Electrolyte Leakage and Chlorophyll Content

Three-week-old WT and *CmTCP13* transgenic lines were watered with 200 mM NaCl, and leaves were collected to measure electrolyte leakage and chlorophyll levels 7 days post-treatment. The electrical conductivity of the leaf tissue was measured using a DDS-307 conductivity meter, as previously reported [65]. The chlorophyll content was determined using a slightly modified method [66]. Briefly, 100 mg (fresh weight) of the rosette leaves of the WT and transgenic lines were collected and immersed in 5 mL 95% ethanol for 48 h in the dark, after which the chlorophyll content was determined using a DU 800 UV/Vis spectrophotometer (Beckman Coulter, Indianapolis, IN, USA) by scanning at 663 and 645 nm.

### 4.8. qRT-PCR Analyses

Total RNA was extracted from the leaves of the salinity-stressed chrysanthemum and *Arabidopsis* plants using an RNA extraction kit (Huayueyang, Beijing, China). First-strand cDNA was synthesized using a PrimeScript^TM^ RT reagent kit (TaKaRa Bio Inc., Shiga, Japan). A LightCycler 96 Real-Time PCR System (Roche, Basel, Switzerland) was used for qRT-PCR experiments using an SYBR Premix Ex Taq II kit (TaKaRa Bio Inc., Shiga, Japan). The *Chrysanthemum CmEF1a* and *Arabidopsis AtActin2* genes were used as the endogenous controls. Each sample was evaluated based on three biological and three technical replicates. The relative transcript abundances were determined using the 2^−ΔΔCT^ method [67]. All primers used for qRT-PCR are listed in Table S1.

### 4.9. Statistical Analyses

All statistical analyses were conducted using SPSS v25.0 software (SPSS Inc., Chicago, IL, USA). Significantly different trait values were determined using Duncan’s multiple range test. Statistical significance was considered at *p* < 0.05.

## Figures and Tables

**Table 1:** *Cis*-acting element analysis of *CmTCP13* gene promoter.

Motif Name	Motif Sequence	Function	No.	Position
ABRE	ACGTG/CACGTG/GCAACGTGTC	Abscisic acid response element	5	1704, −2123, −1780, −1778, 1781
ARE	AAACCA	Anaerobic-responsive element	6	195, −1655, −1191, 1903, −871, 1536
ATC-motif	AGTAATCT	Light-responsive element	1	1722
AuxRR-core	GGTCCAT	Auxin response element	1	555
Box 4	ATTAAT	Light-responsive element	1	905
Box III	atCATTTTCACt	Protein binding site	1	291
CGTCA-motif	CGTCA	MeJA-responsive element	2	75, −1715
ERE	ATTTTAAA	Ethylene-responsive element	1	636
G-box	CACGTT/CACGTG/ACACGTGGC/TACGTG	Light-responsive element	4	−1703, −1780, 1799, −2123
GA-motif	ATAGATAA	Light-responsive element	1	606
GATA-motif	AAGATAAGATT/AAGGATAAGG	Light-responsive element	3	−211, 2302, −2164
GT-1 motif	GAAAAA	Salinity stress-inducible element	7	95, 425, 1385, 2048, −294, −1145, −2342
HD-Zip 3	GTAAT(G/C)ATTAC	Protein binding site	1	701
I-box	AGATAAGG/cCATATCCAAT	Part of a light-responsive element	2	239, −1832
LTR	CCGAAA	Low-temperature response element	1	423
MBS	CAACTG	MYB binding site involved in drought-inducibility	1	1267
MYB	TAACCA/CAACCA/CAACAG	MYB/YB recognition site	5	−1027, −2065, −1844, −1840, −2003
MYC	CATGTG	MYC/YC recognition site	1	1283
P-box	CCTTTTG	Gibberellin-responsive element	1	695
TCA-element	CCATCTTTTT	Salicylic acid-responsive element	1	289
TGA-element	AACGAC	Auxin-responsive element	1	−44
TGACG-motif	TGACG	MeJA-responsive element	2	5, −5
WUN-motif	AAATTTCCT	Wound-responsive element	1	−111
Circadian	CAAAGATATC	Circadian regulation element	1	−214

Abbreviations: No., Number; MeJA, Methyl jasmonate; HD-Zip 3, Homeodomain leucine zipper protein 3.

## Data Availability

Data are contained within the article and Supplementary Materials.

## Supplementary Materials

The following supporting information can be downloaded at https://www.mdpi.com/article/10.3390/plants13152118/s1, Table S1: List of primers used in this study. Figure S1: Expression of endogenous *AtTCP13* in the WT and *CmTCP13*-overexpressing transgenic *Arabidopsis* plants as assessed by qRT-PCR.
