# Supplementary figures and images for: Heterologous Expression of Chrysanthemum TCP Transcription Factor CmTCP13 Enhances Salinity Tolerance in Arabidopsis
Source: Plants (Basel). 2024 Jul 31;13(15):2118. doi: 10.3390/plants13152118 (PMC11313808; doi:10.3390/plants13152118)

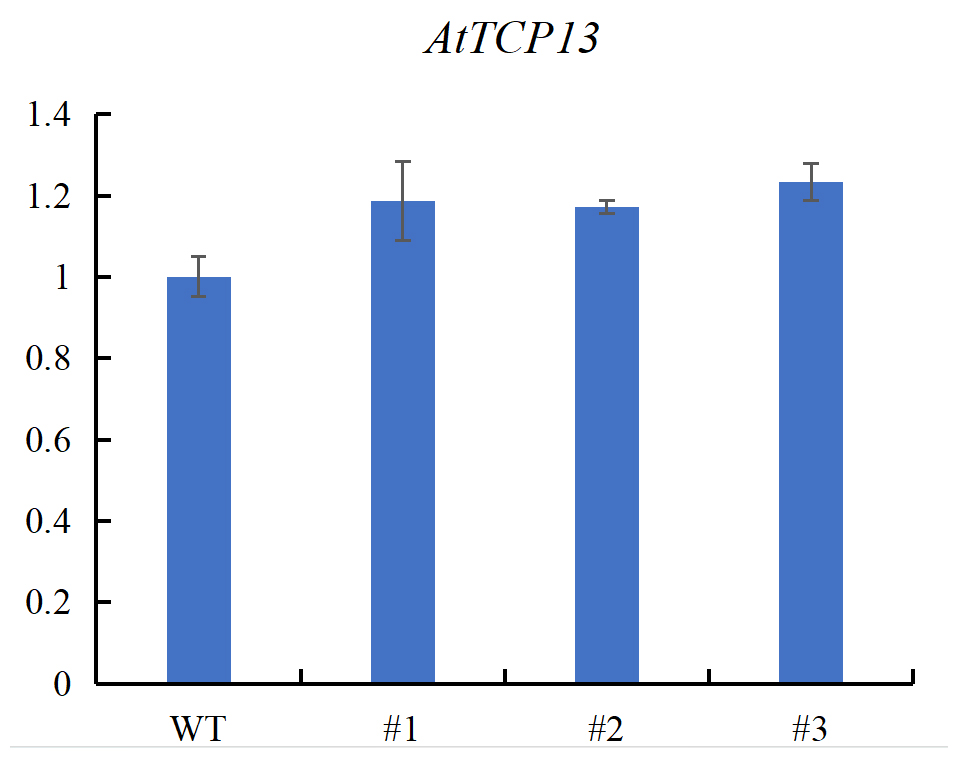

Supplement: Supplementary file 1 [file plants-13-02118-s001.zip › Figure S1.jpg]
